# The “sensor domains” of plant NLR proteins: more than decoys?

**DOI:** 10.3389/fpls.2015.00134

**Published:** 2015-03-05

**Authors:** Chih-Hang Wu, Ksenia V. Krasileva, Mark J. Banfield, Ryohei Terauchi, Sophien Kamoun

**Affiliations:** ^1^The Sainsbury Laboratory, Norwich Research ParkNorwich, UK; ^2^The Genome Analysis Centre, Norwich Research ParkNorwich, UK; ^3^Department of Biological Chemistry, John Innes Centre, Norwich Research ParkNorwich, UK; ^4^Iwate Biotechnology Research CenterKitakami, Japan

**Keywords:** NLR protein pairs, integrated decoy, pathogen recognition, plant immunity, *Arabidopsis thaliana*, rice, sensor domain, decoy

Our conceptual and mechanistic understanding of how plant nucleotide-binding leucine-rich repeat (NLR or NB-LRR) proteins perceive pathogens continues to advance. NLRs are intracellular multidomain proteins that recognize pathogen-derived effectors either directly or indirectly (Jones and Dangl, 2006; Van Der Hoorn and Kamoun, 2008; Dodds and Rathjen, 2010; Cesari et al., 2014). In the direct model, the NLR protein binds a pathogen effector or serves as a substrate for the effector's enzymatic activity. In the indirect model, the NLR recognizes modifications of additional host protein(s) targeted by the effector. Such intermediate host protein(s) are often called effector targets (ETs). However, given that effectors can act on multiple host targets, the specific protein that mediates recognition by the NLR may not be the effector's operative target and may have evolved to function as a decoy dedicated to pathogen detection. This “decoy” model contrasts with the “guard” model in which the NLR perceives the effector via its action on its operative target (Van Der Hoorn and Kamoun, 2008).

In a recent article, Cesari et al. (2014) elegantly synthesized the literature to propose a novel model of how NLRs recognize effectors termed the “integrated decoy” hypothesis. Based on new data from several pathosystems, it appears that some NLRs recognize pathogen effectors through extraneous domains that have evolved by duplication of an ET followed by fusion into the NLR. This NLR-integrated domain mimics the effector binding/substrate property of the original ET to enable pathogen detection. In addition, these “receptor” or “sensor” NLRs typically partner with NLR proteins with a classic architecture that function as signaling partners required for the resistance response (Eitas and Dangl, 2010; Cesari et al., 2013, 2014; Williams et al., 2014).

Here, we expand on the Cesari et al. (2014) model and introduce the possibility that NLR-integrated domains do not have to be decoys (as in defective mimics) of the effector's operative target. Indeed, in addition to binding effectors or serving as their substrates, operative targets carry a biochemical activity that is modulated by the effector. The perturbation of this activity by the effector leads to effector-triggered susceptibility, an activity often related to immunity (Boller and He, 2009; Dodds and Rathjen, 2010; Win et al., 2012). Clearly NLR-integrated domains must retain the “sensor” activity of the ancestral ET, but they could also retain their biochemical activity, continuing to function in the effector-targeted pathway even as an extraneous domain within a classic NLR architecture. At present, this possibility cannot be discounted given that the biochemical activities of the ancestral ETs and their NLR-integrated counterparts are generally unknown. Additionally, when NLR-fusions occurred recently, there may not have been enough time for the integrated ET to lose its original function and evolve into a decoy. We therefore propose to refer to the extraneous domains of classic NLR proteins described by Cesari et al. (2014) as sensor domains (SD), a term that is agnostic to any potential biochemical activities of the integrated module.

How to test whether or not SDs are decoys? We propose a straightforward genetic test that can reject the decoy hypothesis. Isogenic plants either carrying or lacking the NLR-SD can be challenged with a pathogen strain that lacks the matching avirulence effector (Figure 1). There are several possible outcomes. If the NLR-SD isogenic lines do not differ in their response to the pathogen without the matching effector, the result is inconclusive and the null decoy hypothesis cannot be rejected. If the presence of NLR-SD without the known matching effector shows higher levels of resistance, and there are no signs of typical effector-triggered immunity, then the SD is likely to have retained the ET biochemical activity and contributes to basal immunity in a manner analogous to the ancestral ET. An even more interesting result would be if in the absence of the matching effector, the NLR-SD line is more susceptible as has been shown for several ETs (Van Schie and Takken, 2014). In this scenario, another (unrecognized) effector might still be targeting the original biochemical activity of the SD domain. It would be conceptually fascinating if an NLR that functions as a resistance (R) gene against certain strains of a pathogen becomes a susceptibility (S) gene when exposed to other strains. Once again, this concept emphasizes how the outcome of plant-pathogen interactions is so critically dependent on the genotypes of the interacting organisms—a gene that has a certain impact in a particular genetic combination can have the exact opposite effect in another (Jones and Dangl, 2006; Van Der Hoorn and Kamoun, 2008; Dodds and Rathjen, 2010; Win et al., 2012).

**Figure 1:**
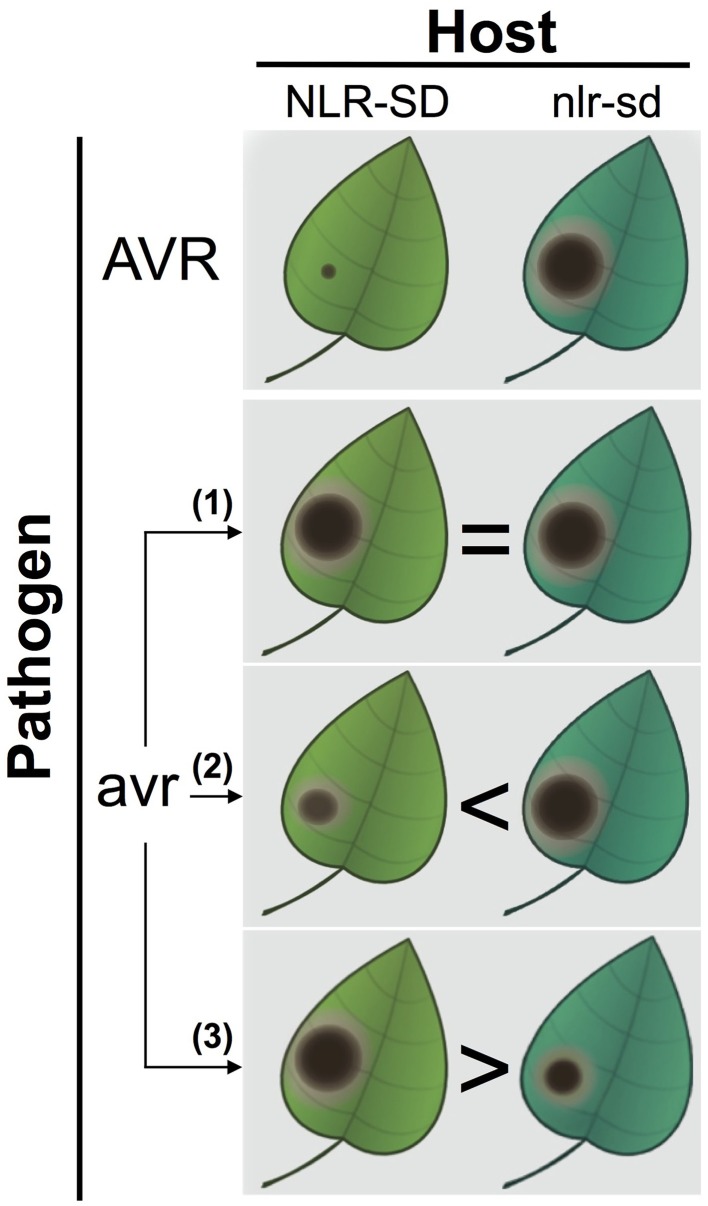
**A genetic test to inform whether NLR-SD proteins have retained a biochemical activity independent of perception of an avirulence effector**. In the top panel, isogenic plants either carrying or lacking the NLR-SD display differential resistance to a pathogen strain carrying the AVR (avirulence) effector (top panel, NLR-SD plants displaying full resistance to the avirulent pathogen strain). To challenge the decoy hypothesis, the differential NLR-SD lines are challenged with a pathogen strain that lacks the AVR effector (avr) and is isogenic to the AVR strain. In these experiments, three outcomes can be expected. (1) No differences between the NLR-SD lines are observed resulting in inconclusive results—the null decoy hypothesis cannot be rejected. The reason the result is inconclusive is because it is now accepted that effectors have other activities than suppression of immunity (nutrition, development, epigenetics etc.), and therefore the targeted host proteins do not necessarily modulate susceptibility/resistance phenotypes. (2) The plants carrying the NLR-SD are more resistant to the avr pathogen strain that lacks the AVR effector. (3) The plants carrying the NLR-SD are more susceptible to the avr pathogen strain that lacks the AVR effector. In these two cases, the SD is likely to have retained the biochemical activity of its ancestral host protein and the decoy hypothesis can be rejected. In scenario (2), the higher levels of resistance to the avr pathogen conferred by the NLR-SD are consistent with a role of the SD in basal immunity analogous to the ancestral target. In scenario (3), however, the NLR-SD is more susceptible to its isogenic line possibly because the SD is targeted by another (unrecognized) effector. In such a case, the NLR-SD resistance (*R*) gene becomes a susceptibility (*S*) gene depending on the genotype of the pathogen it is challenged with.

Our goal is not to engage in an exercise in semantics. However, we wish to avoid conceptually restrictive terminology and urge the plant-microbe interactions community to test a rich spectrum of models and hypotheses. The proposed sensor domain terminology would accommodate this breadth of ideas. Ultimately, it may very well turn out that the majority, if not all, of the NLR integrated domains have lost their biochemical activities and have evolved into decoys. Also, it is possible that the sensor domain has already evolved into a decoy prior to recombination into a NLR. Nonetheless, further genetic and biochemical experiments are required to determine whether sensor domains of NLR-SDs are decoys or biochemically functional duplicates of their ancestral ETs.

## Conflict of interest statement

The authors declare that the research was conducted in the absence of any commercial or financial relationships that could be construed as a potential conflict of interest.
